# Assessment of US Public School District Policies for Pandemic Preparedness and Implications for COVID-19 Response Activities

**DOI:** 10.1017/dmp.2020.496

**Published:** 2020-12-22

**Authors:** Cassandra A. Kersten, Allison T. Chamberlain, Sherry Everett Jones, Amra Uzicanin, Faruque Ahmed

**Affiliations:** 1 Division of Global Migration and Quarantine, Centers for Disease Control and Prevention, Atlanta, Georgia USA; 2 Department of Epidemiology, Rollins School of Public Health, Emory University, Atlanta, Georgia USA; 3 Booz Allen Hamilton, Falls Church, VA, USA; 4 Division of Adolescent and School Health, Centers for Disease Control and Prevention, Atlanta, Georgia USA

**Keywords:** school closure, pandemic preparedness, COVID-19, influenza, influenza pandemic

## Abstract

**Objectives:**

To describe school district preparedness for school closures and other relevant strategies before the coronavirus disease 2019 (COVID-19) pandemic.

**Methods:**

A stratified random sample of 957 public school districts from the 50 US states and the District of Columbia were surveyed between October 2015 and August 2016. The response rates for the questionnaires were as follows: Healthy and Safe School Environment, Crisis Preparedness Module (60%; *N* = 572), Nutrition Services (63%; *N* = 599), and Health Services (64%; *N* = 613). Data were analyzed using descriptive and regression techniques.

**Results:**

Most school districts had procedures that would facilitate the implementation of school closures (88.7%). Fewer districts had plans for ensuring continuity of education (43.0%) or feeding students during closure (33.8%). The prevalence of continuity of education plans was lower in the Midwest than the Northeast (adjusted prevalence ratio [aPR] = 0.68; 95% confidence interval [CI]: 0.51-0.90). Presence of plans for feeding students was higher in high-poverty than low-poverty districts (aPR = 1.41; 95% CI: 1.01-1.99) and in large districts than small districts (aPR = 2.06; 95% CI: 1.37-3.09).

**Conclusions:**

Understanding factors associated with having comprehensive emergency plans could help decision makers to target assistance during the current COVID-19 pandemic and for future planning purposes.

Preemptive school closure, or school dismissal where a school stays open for staff members while the children stay home, may be 1 of several recommended nonpharmaceutical interventions (NPIs) during a pandemic to dampen and delay the epidemic peak and “buy time” for the production of effective countermeasures or vaccines.^1-7^ “Flattening the curve” can also prevent overwhelming the health-care system.^8^ Multiple systematic literature reviews of the effectiveness of school closures to reduce influenza transmission in schools and surrounding communities have been performed by different groups since the 2009 influenza A (H1N1) pandemic; they all concurred on the effectiveness of pre-emptive, coordinated, school closures in delaying the peak during a severe influenza pandemic.^2,3^ When used to mitigate pandemics, school closures are more effective when they are implemented before widespread viral transmission has occurred among students and staff.^2,6^ Implementation of a combination of NPIs can have a more pronounced effect.^4^

School closures entail various social and economic implications.^5^ In a survey conducted in 2017 following an 8-d closure in rural Illinois, 17% of families indicated challenges, including uncertainty about closure duration, childcare, and lost pay.^6^ In another investigation following a 4.5-d closure in Colorado in 2013, 20% of households reported challenges, including lost pay, uncertainty about the duration, and concerns about the loss of free school meals.^7^ That investigation also assessed the perceived difficulty of a 1-mo closure; 29% believed it would impose a minor to major problem, and 9% were unsure whether it would cause a problem.^7^

During response to influenza pandemics and other emergencies, decisions regarding school closures are typically made by state and local officials.^5^ The disruptive nature of school closures and lack of standardized procedures highlights the importance of prepandemic planning.^5^

School districts have been identified as a key partner in a community’s ability to effectively implement emergency response related to infectious disease outbreaks or pandemics.^8^ However, few studies have examined the policies related to pandemic preparedness that are in place in school districts. Using data from the 2016 School Health Policies and Practices Study (SHPPS), this analysis aims to describe and evaluate school district policies that were in effect before the coronavirus disease 2019 (COVID-19) pandemic that may have impacted the logistics of physically closing schools and the ability of school districts to balance factors related to continuity of education and nutrition services during an extended closure. Although school districts may have updated their policies since SHPPS was conducted in 2016, the SHPPS 2016 national-level data can provide useful insights regarding school readiness when the COVID-19 pandemic began in the United States in the spring of 2020.

## Methods

### Subjects and Instruments

Data for this study are derived from the SHPPS. Public school districts from the 50 US states and the District of Columbia were selected using a stratified, random sample (*N* = 957). A more detailed description of SHPPS 2016 methods is available elsewhere.^9^ The sampling frame was derived from the October 2015 Market Data Retrieval (MDR) database,^10^ a commercial database that identifies US schools and school districts, and a wide array of demographic characteristics about them. The SHPPS survey questionnaires were sent to 957 eligible school districts for completion between October 2015 and August 2016.^9^

SHPPS 2016 included the following questionnaires that were examined for this study: (1) Healthy and Safe School Environment, (2) Nutrition Services, and (3) Health Services.^9^ The response rates and sample sizes for the 3 questionnaires were as follows: Healthy and Safe School Environment, Crisis Preparedness Module (60%; *N* = 572), Nutrition Services (63%; *N* = 599), and Health Services (64%; *N* = 613). A description of the districts that participated in each questionnaire is presented in Supplementary Material Table S1. The 2016 SHPPS study was approved as exempt research by ICF International’s institutional review board and CDC’s Human Research Protection Office.

### Procedure

The SHPPS questions included in this study are shown in Supplementary Material Table S2 and are all dichotomous, yes/no questions. SHPPS 2016 data were linked with MDR data to identify each district’s Census region (Northeast, South, Midwest, and West), metropolitan status (urban, suburban, town, rural), and enrollment size (small [<2500 students], medium [2500-9999 students], and large [≥10,000 students]).^11^ District poverty level was calculated by MDR by creating a ratio of the children in a district from families below the poverty line to all children in the district aggregated across all schools in the districts and coded as 0-15.9%, 16.0-30.9%, and 31.0% or higher, indicating the percentage of children in the district from families below the poverty line.

### Analyses

Analyses were conducted using SAS-callable SUDAAN to account for both the stratified random sample design and weighting. Wald chi-squared tests were used to assess differences in district policies by district demographic characteristics. When the Wald chi-squared tests were significant, pairwise comparisons were conducted using a t-test for proportions. Logistic regression was used in SUDAAN to compute adjusted prevalence ratios (aPR) by means of predicted marginal standardization to examine the association between demographic characteristics and the variables of interest. Variables included as confounders were determined by means of literature and field expertise, and included urbanicity, district poverty level, US Census region, and district enrollment size.

## Results

### Overall Pandemic Preparedness

Nationwide, most school districts (94.6%) reported having a comprehensive district-level plan to address crisis preparedness, response, and recovery in the event of a natural disaster or other emergency or crisis. Fewer districts indicated that their district’s crisis preparedness, response, and recovery plan included procedures for implementing unplanned school dismissal or school closure (88.7%) or had procedures for responding to pandemic influenza or other infectious disease outbreaks (73.6%) (Table 1). Having a district plan that included procedures for pandemic influenza or other infectious disease outbreaks varied by metropolitan status and district enrollment size, with rural districts significantly less likely to have these procedures than districts in urban or suburban areas. Similarly, large districts were significantly more likely to have plans than small districts.

**Table 1.** Plans and procedures related to unplanned school closures or dismissals among US school districts, SHPPS 2016

District characteristics	District has a comprehensive plan to address crisis preparedness, response, and recovery % (95% CI)	District has procedures for implementing unplanned school dismissal or school closure % (95% CI)	District plan includes procedures for responding to pandemic influenza or other infectious disease outbreaks % (95% CI)
No. of observations	567	567	559
Total	94.6 (92.3–96.2)	88.7 (85.5–91.2)	73.6 (69.7–77.2)
Metropolitan status			
Urban	96.3 (77.6–99.5)	94.1 (78.1–98.6)	90.4 (73.5–96.9)
Suburban	99.1 (93.8-99.9)	91.1 (83.6–95.4)	82.1 (73.5–88.4)
Town	96.4 (89.2–98.9)	88.4 (78.9–94.0)	80.0 (69.7–87.4)
Rural	91.9 (88.1–94.6)	87.2 (82.8–90.7)	66.2 (60.7–71.3)
*P*-value	0.002	0.43	<0.001
District poverty (%)^a^			
0–15.9	96.0 (92.1–98.0)	89.4 (84.2–93.0)	72.4 (66.1–77.8)
16–30.9	93.2 (89.1–95.8)	87.9 (82.9–91.6)	73.1 (66.9–78.5)
31+	95.0 (86.6–98.3)	89.2 (78.4–94.9)	79.6 (68.4–87.6)
*P*-value	0.45	0.89	0.43
US Census region^b^			
Northeast	95.6 (90.4–98.0)	91.9 (85.5–95.7)	77.5 (69.0–84.2)
Midwest	96.3 (93.1–98.1)	89.8 (84.9–93.3)	70.1 (63.5–75.9)
South	95.4 (90.6–97.8)	93.0 (87.6–96.1)	77.6 (70.2–83.7)
West	89.1 (79.5–94.5)	77.8 (66.5–86.1)	71.4 (59.6–80.8)
*P*-value	0.34	0.049	0.30
District enrollment size			
Small (<2,500)	93.3 (90.1–95.5)	88.0 (84.0–91.1)	70.3 (65.4–74.8)
Medium (2,500–9,999)	97.2 (92.6–99.0)	89.6 (82.7–93.9)	76.6 (68.5–83.2)
Large (≥10,000)	97.5 (83.8–99.7)	92.0 (76.3–97.6)	93.0 (80.7–97.7)
*P*-value	0.10	0.70	<0.001

Data Source: School Health Policies and Practices Study (SHPPS), Centers for Disease Control and Prevention, 2016.

Note: No. of observations are unweighted; Percentages are weighted, unadjusted estimates.

a Percentage of the children in a district from families below the poverty line to all children in the district aggregated across all schools in the districts.

b Northeast: Connecticut, Maine, Massachusetts, New Jersey, New Hampshire, New York, Pennsylvania, Rhode Island, and Vermont; Midwest: Illinois, Indiana, Iowa, Kansas, Michigan, Minnesota, Missouri, Nebraska, North Dakota, Ohio, South Dakota, and Wisconsin; South: Alabama, Arkansas, Delaware, District of Columbia, Florida, Georgia, Kentucky, Louisiana, Maryland, Mississippi, North Carolina, Oklahoma, South Carolina, Tennessee, Texas, Virginia and West Virginia; West: Alaska, Arizona, California, Colorado, Hawaii, Idaho, Montana, Nevada, New Mexico, Oregon, Utah, Washington, and Wyoming.

### Factors Influencing the Ability of US School Districts to Monitor the Effect of Seasonal or Pandemic Infectious Diseases

The ability of school districts and associated health departments to monitor the effects of seasonal or pandemic infectious diseases varied by geodemographic factors (Table 2). Approximately one-third (33.7%) of school districts required schools to close or dismiss all students at a specified level of absent students or staff. A total of 86.1% of districts had adopted a policy stating that schools will obtain and keep reasons for absence in student records, and 91.6% recommended that schools within their district use an electronic system for reporting student absenteeism. The policy that schools record reasons for student absences varied significantly by region, with districts in the South and Midwest more likely to collect this information than districts in the Northeast and West. District policies recommending use of an electronic reporting system were more prevalent in urban and suburban districts than rural districts, and in large than in small districts.

**Table 2.** Ability of US school districts to monitor the effect of seasonal or pandemic infectious diseases, SHPPS 2016

District characteristics	District requires schools to close or dismiss all students when the percentage of absent students or staff reaches a specified level % (95% CI)	District has adopted a policy that schools will obtain and keep the reasons for absence in any type of student record % (95% CI)	District recommends that schools use a specified electronic system for reporting student attendance or absenteeism % (95% CI)	District or local health department has real-time access to data on student attendance or absenteeism for all schools in the district % (95% CI)	District requires schools to submit reasons for student absences to the school district or local health department % (95% CI)
No. of observations	532	592	572	570	562
Total	33.7 (29.6–38.0)	86.1 (82.8–88.8)	91.6 (88.8–93.8)	75.1 (71.2–78.7)	53.0 (48.7–57.3)
Metropolitan status					
Urban	30.4 (16.5–49.1)	88.3 (73.6–95.4)	97.9 (87.3–99.7)	83.2 (64.2–93.2)	68.1 (49.8–82.1)
Suburban	26.2 (18.2–36.0)	86.9 (79.4–92.0)	95.9 (90.5–98.3)	74.4 (65.4–81.7)	64.4 (54.4–73.2)
Town	32.8 (23.4–43.7)	83.0 (73.5–89.6)	90.6 (80.9–95.6)	70.7 (60.3–79.2)	54.8 (44.0–65.1)
Rural	37.6 (32.1–43.4)	86.6 (82.1–90.1)	89.3 (85.2–92.4)	76.2 (70.9–80.7)	45.8 (40.2–51.6)
*P*–value	0.20	0.84	0.009	0.51	0.002
District poverty (%)^a^					
0 – 15.9	26.2 (20.8–32.6)	82.7 (77.2–87.1)	91.0 (86.5–94.0)	77.6 (71.7–82.6)	50.7 (44.3–57.2)
16 – 30.9	41.5 (34.8–48.4)	88.8 (83.7–92.4)	91.5 (86.7–94.7)	72.9 (66.5–78.5)	55.0 (48.2–61.7)
31+	32.7 (22.5–44.9)	91.9 (82.4–96.4)	94.5 (85.3–98.1)	75.4 (64.2–84.0)	57.2 (45.2–68.4)
*P*–value	0.005	0.06	0.57	0.52	0.53
US Census region^b^					
Northeast	26.2 (18.8–35.2)	79.5 (71.5–85.7)	91.8 (84.0–96.0)	81.0 (73.0–87.0)	52.8 (43.8–61.6)
Midwest	36.4 (29.7–43.5)	88.9 (83.8–92.6)	89.0 (84.0–92.6)	75.6 (69.0–81.1)	56.9 (49.8–63.7)
South	39.0 (31.4–47.1)	92.3 (87.1–95.5)	93.9 (89.0–96.7)	69.1 (61.4–75.9)	50.3 (42.4–58.1)
West	29.8 (19.6–42.6)	80.1 (68.6–88.1)	93.6 (84.1–97.6)	75.1 (63.0–84.2)	49.1 (36.9–61.4)
*P*–value	0.12	0.007	0.36	0.16	0.57
District enrollment size					
Small (<2,500)	37.2 (32.0–42.6)	85.5 (81.3–88.9)	89.0 (85.1–91.9)	73.4 (68.5–77.8)	49.3 (43.9–54.6)
Medium (2,500–9,999)	26.8 (19.4–35.7)	86.6 (79.7–91.4)	95.9 (90.0–98.4)	78.5 (70.5–84.7)	62.7 (54.1–70.6)
Large (≥10,000)	28.1 (16.9–43.0)	88.5 (76.9–94.7)	98.6 (91.7–99.8)	77.7 (62.3–88.1)	52.2 (37.4–66.6)
*P*–value	0.08	0.81	<0.001	0.47	0.03

Data Source: School Health Policies and Practices Study (SHPPS), Centers for Disease Control and Prevention, 2016.

Note: No. of observations are unweighted; Percentages are weighted, unadjusted estimates.

a Percentage of the children in a district from families below the poverty line to all children in the district aggregated across all schools in the districts.

b Northeast: Connecticut, Maine, Massachusetts, New Jersey, New Hampshire, New York, Pennsylvania, Rhode Island, and Vermont; Midwest: Illinois, Indiana, Iowa, Kansas, Michigan, Minnesota, Missouri, Nebraska, North Dakota, Ohio, South Dakota, and Wisconsin; South: Alabama, Arkansas, Delaware, District of Columbia, Florida, Georgia, Kentucky, Louisiana, Maryland, Mississippi, North Carolina, Oklahoma, South Carolina, Tennessee, Texas, Virginia and West Virginia; West: Alaska, Arizona, California, Colorado, Hawaii, Idaho, Montana, Nevada, New Mexico, Oregon, Utah, Washington, and Wyoming.

Three-quarters (75.1%) of school districts indicated that either their district or the local health department, or both, had real-time access to data on student absenteeism for all schools in the district (Table 2). Fewer (53.0%) districts reported that schools are required to submit information to the school district or to the local health department on the reasons for student absences. This requirement varied significantly by metropolitan status, with urban and suburban districts being more likely to have this policy than rural districts; it also varied by district size, with large and medium districts being more likely to have this policy than small districts (Table 2).

### Factors Related to Continuity of Education and Food and Nutrition Services

Most districts (93.6%) reported that their district’s crisis preparedness, response, and recovery plan included mechanisms for communicating with parents or guardians of students (Table 3). Approximately one-third (33.8%) of districts reported having a district-level plan for feeding students who rely on the school meal programs in the event of an unplanned school dismissal or school closure; this differed by metropolitan status and district enrollment size, with large and urban districts most likely to have plans for feeding students (Table 3). Districts with a high poverty level were more likely to have plans for feeding students during unplanned school dismissal/closure than schools with a low district poverty level after controlling for urbanicity, US Census region, and district enrollment size (aPR = 1.41; 95% CI: 1.01-1.99) (Table 4). Large districts were also much more likely to have plans for feeding students than small districts after controlling for urbanicity, district poverty level, and US Census region (aPR = 2.06; 95% CI: 1.37-3.09) (Table 4).

**Table 3.** US school district plans for continuity of education and nutritional services during unplanned school closures or dismissals, SHPPS 2016

District characteristics	District has plans for feeding students during unplanned school dismissal/closure % (95% CI)	District plans include procedures for ensuring the continuity of education % (95% CI)	District plan includes a mechanism for communicating with parents or guardians of students % (95% CI)
No. of observations	526	554	565
Percentage of districts	33.8 (29.7–38.2)	43.0 (38.8–47.4)	93.6 (91.1–95.4)
Metropolitan status			
Urban	57.4 (39.7–73.4)	48.2 (31.7–65.0)	94.1 (78.1–98.6)
Suburban	38.5 (28.9–49.1)	49.9 (40.2–59.7)	99.1 (93.7–99.9)
Town	39.1 (28.9–50.4)	44.2 (33.6–55.5)	95.1 (87.4–98.2)
Rural	27.3 (22.4–32.7)	39.2 (33.9–44.8)	90.8 (86.8-93.6)
*P*-value	0.003	0.25	<0.001
District poverty (%)^a^			
0 – 15.9	34.3 (27.9–41.4)	42.6 (36.0–49.4)	95.6 (91.7–97.7)
16 – 30.9	29.8 (23.9–36.5)	41.1 (34.8–47.7)	91.9 (87.5–94.8)
31+	46.2 (35.2–57.6)	47.3 (36.0–58.9)	93.1 (84.6–97.0)
*P*-value	0.06	0.67	0.27
US Census region^b^			
Northeast	34.0 (25.6-43.4)	52.0 (42.6–61.3)	95.5 (90.3–98.0)
Midwest	31.4 (24.8–38.9)	33.5 (27.3–40.3)	95.1 (91.6–97.2)
South	38.8 (31.0–47.3)	52.2 (44.0–60.3)	94.2 (89.2-97.0)
West	31.5 (21.6–43.4)	40.0 (28.8-52.3)	87.8 (78.0-93.6)
*P*-value	0.57	0.001	0.33
District enrollment size			
Small (<2,500)	27.5 (22.9–32.8)	41.7 (36.7–46.9)	92.4 (89.1–94.7)
Medium (2,500 – 9,999)	39.8 (31.4–48.9)	40.6 (32.0–49.8)	96.2 (90.9–98.5)
Large (≥10,000)	61.2 (44.7–75.4)	62.1 (45.4–76.3)	95.9 (84.3–99.0)
*P*-value	<0.001	0.07	0.19

Data Source: School Health Policies and Practices Study (SHPPS), Centers for Disease Control and Prevention, 2016.

Note: No. of observations are unweighted; Percentages are weighted, unadjusted estimates.

a Percentage of the children in a district from families below the poverty line to all children in the district aggregated across all schools in the districts.

b Northeast: Connecticut, Maine, Massachusetts, New Jersey, New Hampshire, New York, Pennsylvania, Rhode Island, and Vermont; Midwest: Illinois, Indiana, Iowa, Kansas, Michigan, Minnesota, Missouri, Nebraska, North Dakota, Ohio, South Dakota, and Wisconsin; South: Alabama, Arkansas, Delaware, District of Columbia, Florida, Georgia, Kentucky, Louisiana, Maryland, Mississippi, North Carolina, Oklahoma, South Carolina, Tennessee, Texas, Virginia and West Virginia; West: Alaska, Arizona, California, Colorado, Hawaii, Idaho, Montana, Nevada, New Mexico, Oregon, Utah, Washington, and Wyoming.

**Table 4.** aPRs^a^ of US school districts having plans for continuity of education and nutritional services during unplanned school closures or dismissals, SHPPS 2016

District characteristics	District has plans for feeding students during unplanned school dismissal/closure aPR (95% CI)	District plans include procedures for ensuring the continuity of education aPR (95% CI)	District plan includes a mechanism for communicating with parents or guardians of students aPR (95% CI)
Metropolitan status			
Urban	1.35 (0.74–2.45)	1.04 (0.60–1.80)	1.03 (0.94–1.13)
Suburban	1.09 (0.75–1.59)	1.21 (0.90–1.62)	1.08 (1.03–1.14)
Town	1.29 (0.93–1.80)	1.17 (0.89–1.55)	1.04 (0.98–1.10)
Rural	Ref	Ref	Ref
District poverty (%)^b^			
0 - 15.9	Ref	Ref	Ref
16 - 30.9	0.91 (0.67–1.24)	0.99 (0.78-1.24)	0.98 (0.93–1.03)
31+	1.41 (1.01–1.99)	1.06 (0.76–1.47)	0.99 (0.92-1.06)
US Census region^c^			
Northeast	Ref	Ref	Ref
Midwest	0.92 (0.65–1.28)	0.68 (0.51–0.90)	1.01 (0.95–1.07)
South	0.93 (0.63–1.37)	1.01 (0.75–1.36)	1.00 (0.95–1.06)
West	0.81 (0.53–1.26)	0.77 (0.53–1.12)	0.94 (0.85–1.03)
District enrollment size			
Small (<2,500)	Ref	Ref	Ref
Medium (2,500–9,999)	1.32 (0.94–1.85)	0.86 (0.64–1.16)	1.01 (0.95–1.08)
Large (≥10,000)	2.06 (1.37–3.09)	1.36 (0.92–2.01)	1.01 (0.94–1.09)

Data Source: School Health Policies and Practices Study (SHPPS), Centers for Disease Control and Prevention, 2016.

a Logistic regression models included metropolitan status, district poverty level, US Census region, and district enrollment size.

b Percentage of the children in a district from families below the poverty line to all children in the district aggregated across all schools in the districts.

c Northeast: Connecticut, Maine, Massachusetts, New Jersey, New Hampshire, New York, Pennsylvania, Rhode Island, and Vermont; Midwest: Illinois, Indiana, Iowa, Kansas, Michigan, Minnesota, Missouri, Nebraska, North Dakota, Ohio, South Dakota, and Wisconsin; South: Alabama, Arkansas, Delaware, District of Columbia, Florida, Georgia, Kentucky, Louisiana, Maryland, Mississippi, North Carolina, Oklahoma, South Carolina, Tennessee, Texas, Virginia and West Virginia; West: Alaska, Arizona, California, Colorado, Hawaii, Idaho, Montana, Nevada, New Mexico, Oregon, Utah, Washington, and Wyoming.

Almost half (43.0%) of school districts had plans for ensuring continuity of education during unplanned school closure, although this was least common in school districts in the Midwest (Table 3). Midwest schools were 32% less likely to have plans for ensuring the continuity of education than Northeast schools (aPR = 0.68; 95% CI: 0.51-0.90) after controlling for urbanicity, district poverty level, and district enrollment size (Table 4).

## Discussion

Findings from the 2016 SHPPS Survey indicate that nationwide in 2016, a total of 94.6% of public school districts reported having a comprehensive district-level plan to address crisis preparedness, response, and recovery in the event of a natural disaster or other emergency. However, less than half of the school districts had plans for continuity of education or feeding students during unplanned school closure. Plans for ensuring continuity of education were least likely in the Midwest, and those for feeding students during closure were less common in small school districts but more common in high-poverty school districts.

Few published studies have addressed school district policies on crisis preparedness, response, and recovery. Kruger et al. found in an analysis of SHPPS 2016 data that many school districts were underprepared for emergencies and lacked plans for infectious disease outbreaks.^12^ The current study extends these findings by exploring variables related to decision-making for school closures, including procedures related to school district closure and the ability of public health authorities to monitor reasons for absenteeism, and variables related to the provision of school meals and continuity of education.

Understanding the decision-making process for school closure is important in planning. Absenteeism rates due to influenza-like illness in schools is 1 of the influenza surveillance indicators that could be used for triggering pre-emptive school closures,^5^ but only 53.0% of districts required schools to submit reasons for student absences to the district or local health department. However, because studies of the epidemiology of COVID-19 have found high rates of asymptomatic infection in children,^13^ syndromic surveillance among children may not be helpful in the timely detection of COVID-19 to trigger preemptive school closures or other mitigation measures.^14^

Before the COVID-19 pandemic, it had been widely anticipated that a pandemic influenza threat in the United States would disproportionately affect socially disadvantaged groups and could exacerbate socioeconomic and other race-/ethnicity-related disparities.^15-17^ Addressing such disparities would require sustained community partnerships and involvement.^15,16^ During the COVID-19 pandemic, poverty has been directly associated with COVID-19 mortality rates and COVID-19 has disproportionately affected economically disadvantaged and rural communities.^17,18^ Our findings indicate that only one-third of school districts have plans for feeding students during unplanned school closures, but it is encouraging that higher poverty districts are more likely to have such plans.

During extended school closures, families may experience financial hardship due to missed work as well as food insecurity when free or reduced-price school meals are not available.^19^ Approximately one-third of districts in the United States reported having plans for feeding students during unplanned school closures before the COVID-19 pandemic. With over 95% of schools participating in low-cost or free lunch programs and over 30 million students daily receiving meals through the National School Lunch Program,^20^ the federally assisted school meal program is the second-largest anti-hunger initiative in the United States behind the Supplemental Nutrition Assistance Program.^21^ As such, the loss of free meals has been identified by parents as a top concern during a school closure.^7^ Disruption of school meal programs became an imminent problem during the spring of 2020, when, in response to COVID-19 emergence throughout the United States, nearly all US schools closed following recommendations and mandates from state authorities.^22^ Subsequently, all 50 states have received waivers from the US Department of Agriculture to provide meals in noncongregate settings.^23^ Mechanisms used to provide meals to children have ranged from providing meals at pick-up spots to using existing bus routes to deliver to homes or neighborhood pickup spots.^24^

Only 43.0% of districts in the United States had established procedures for ensuring the continuity of education before COVID-19. A recent RAND working paper indicates that districts without these plans may have greater difficulty making the transition to online learning and supporting student learning during extended closure.^25^ Furthermore, students in high-poverty districts may also have difficulty accessing technology to work on online assignments, particularly when libraries and other community learning spaces are also closed due to stay-at-home orders.^26^ Some innovative solutions to ensuring continuity of education include providing laptops to or sending physical packets home with students,^27,28^ using school buses to provide WiFi,^29^ and providing hotspots or free Internet service to students.^28^

This analysis had some limitations. First, data are limited to public school districts and, thus, may not be applicable to private schools. Within the public school districts, the SHPPS 2016 results found that 90.9% required the periodic review and revision of emergency response plans, indicating that districts may have updated their plans before the COVID-19 pandemic. However, the frequency with which these updates were required was not specified, and likely varied by district.^9^ Additionally, as the data were self-reported, there may have been some level of bias due to overreporting or underreporting or a lack of knowledge by the respondents.^9^ Finally, without having conducted a content analysis of district plans, we do not know the quality or comprehensiveness of the plans that school districts reported having or if they were amended in response to the COVID-19 pandemic.

National guidance for school administrators has been developed for responding to the COVID-19 pandemic.^30^ Further investigation is needed to determine if district plans were effective when used in context of the COVID-19 pandemic and what policies were changed or added by school districts, especially because school closures have likely been much longer than districts originally anticipated and planned for. This information could help refine guidance on best practices to mitigate unintended effects associated with prolonged school closures during this and future emergencies. It would also be useful to determine the types of school districts experiencing difficulty with the implementation and acceptance of closure or with balancing factors related to continuity of education and nutrition services. Although our analysis identified geodemographic factors that were associated with the presence or absence of different school policies before the COVID-19 pandemic, it did not consider the combination of district factors that could directly identify which schools may be more vulnerable than others.

## Conclusions

Findings from SHPPS 2016 suggest many US public school districts may have been underprepared for the school closures that started in March 2020 in response to the COVID-19 pandemic, both in terms of decision-making and continuity of education and nutrition services. The large number of districts without established plans, including basic procedures related to school district closure and the ability to monitor the effect of seasonal or pandemic infectious diseases, may have led to greater difficulty in implementing school closures. Understanding which school districts have comprehensive emergency plans and which do not, could potentially help decision-makers to target assistance both during the current pandemic and for future planning purposes.

## Supplementary material

For supplementary material accompanying this paper visit https://doi.org/10.1017/dmp.2020.496. click here to view supplementary material
