# Supplementary material for: Assessment of US Public School District Policies for Pandemic Preparedness and Implications for COVID-19 Response Activities
Source: Disaster Med Public Health Prep. 2020 Dec 22:1–7. doi: 10.1017/dmp.2020.496 (PMC7985647; doi:10.1017/dmp.2020.496)
Supplement: Supplementary file 1 [file S1935789320004966sup001.docx]

# **Supplementary Tables**

## **TABLE S1. Characteristics of Participating School Districts by Component Questionnaire, SHPPS 2016**

| **District Characteristics** | **Healthy and Safe School Environment**  **(*n* = 572)***  **No. (%)** | **Nutrition Services**  **(*n* = 599)**  **No. (%)** | **Health Services**  **(*n* = 613)**  **No. (%)** |
| --- | --- | --- | --- |
| **Metropolitan Status** |  |  |  |
| Urban | 45 (5.6) | 47 (5.6) | 49 (5.6) |
| Suburban | 109 (21.9) | 113 (23.3) | 123 (23.3) |
| Town | 86 (18.7) | 95 (18.5) | 98 (18.5) |
| Rural | 332 (53.7) | 344 (52.6) | 343 (52.6) |
| Missing | 0 | 0 | 0 |
| **Census Region**^b^ |  |  |  |
| Northeast | 121 (20.4) | 132 (21.4) | 137 (21.4) |
| Midwest | 213 (36.5) | 216 (36.2) | 223 (36.2) |
| South | 162 (24.0) | 173 (23.7) | 178 (23.7) |
| West | 76 (19.0) | 78 (18.7) | 75 (18.7) |
| Missing | 0 | 0 | 0 |
| **District Poverty (%)**^c^ |  |  |  |
| 0 - 15.9 | 230 (43.8) | 243 (44.9) | 258 (45.0) |
| 16 - 30.9 | 249 (42.4) | 251 (41.4) | 255 (41.4) |
| 31+ | 87 (13.8) | 98 (13.7) | 93 (13.6) |
| Missing | 6 | 7 | 7 |
| **District Enrollment Size** |  |  |  |
| Small (<2,500) | 386 (67.4) | 401 (66.6) | 403 (65.9) |
| Medium (2,500 - 9,999) | 128 (24.5) | 138 (25.2) | 142 (25.3) |
| Large (≥10,000) | 58 (8.2) | 60 (8.2) | 68 (8.8) |
| Missing | 0 | 0 | 0 |

Data Source: School Health Policies and Practices Study (SHPPS), Centers for Disease Control and Prevention, 2016.

Note: Numbers are unweighted; percentages are weighted, unadjusted estimates.

^a^Crisis Preparedness, Response, and Recovery module.

^b^Northeast: Connecticut, Maine, Massachusetts, New Jersey, New Hampshire, New York, Pennsylvania, Rhode Island, and Vermont; Midwest: Illinois, Indiana, Iowa, Kansas, Michigan, Minnesota, Missouri, Nebraska, North Dakota, Ohio, South Dakota, and Wisconsin; South: Alabama, Arkansas, Delaware, District of Columbia, Florida, Georgia, Kentucky, Louisiana, Maryland, Mississippi, North Carolina, Oklahoma, South Carolina, Tennessee, Texas, Virginia and West Virginia; West: Alaska, Arizona, California, Colorado, Hawaii, Idaho, Montana, Nevada, New Mexico, Oregon, Utah, Washington, and Wyoming.

^c^Percentage of the children in a district from families below the poverty line to all children in the district aggregated across all schools in the districts.

## **TABLE S2. School Health Policies and Practices Study (SHPPS) Variables of Interest in Assessing US School District Policies for Pandemic Preparedness, 2016**

| **Healthy and Safe School Environment**  **(*n* = 572)^a^** | **Nutrition Services**  **(*n* = 599)** | **Health Services**  **(*n* = 613)** |
| --- | --- | --- |
| Does your district have a comprehensive district-level plan to address crisis preparedness, response, and recovery in the event of a natural disaster or other emergency or crisis situation? | District level plan for feeding students during unplanned school dismissal/closure? | Are schools in your district required to close or dismiss all students when the percentage of absent students or staff reaches a specified level? |
| Does the district plan include procedures for implementing unplanned school dismissal or school closure? |  | Has your district adopted a policy stating that schools will obtain and keep the reasons for absence in any type of student record? |
| Does the district plan include procedures for responding to pandemic influenza (flu) or other infectious disease outbreaks? |  | Does your district recommend that schools use a specified electronic system for reporting student attendance or absenteeism information? |
| Does the district plan include procedures for ensuring the continuity of education  (e.g., online classes, prepackaged assignments) during unplanned school closure? |  | Does your district or local health department have real-time access to student attendance or absenteeism information for all schools in the district? |
| Does the district plan include a mechanism for communicating with parents? |  | Are schools in your district required to submit information to the school district or local health department on the reasons for student absences? |

^a^Crisis Preparedness, Response, and Recovery module.
